# IFITM Proteins Restrict Antibody-Dependent Enhancement of Dengue Virus Infection

**DOI:** 10.1371/journal.pone.0034508

**Published:** 2012-03-30

**Authors:** Ying Kai Chan, I-Chueh Huang, Michael Farzan

**Affiliations:** New England Primate Research Center, Department of Microbiology and Immunobiology, Harvard Medical School, Southborough, Massachusetts, United States of America; University of Hong Kong, Hong Kong

## Abstract

Interferon-inducible transmembrane (IFITM) proteins restrict the entry processes of several pathogenic viruses, including the flaviviruses West Nile virus and dengue virus (DENV). DENV infects cells directly or via antibody-dependent enhancement (ADE) in Fc-receptor-bearing cells, a process thought to contribute to severe disease in a secondary infection. Here we investigated whether ADE-mediated DENV infection bypasses IFITM-mediated restriction or whether IFITM proteins can be protective in a secondary infection. We observed that IFITM proteins restricted ADE-mediated and direct infection with comparable efficiencies in a myelogenous leukemia cell line. Our data suggest that IFITM proteins can contribute to control of secondary DENV infections.

## Introduction

The four serotypes of dengue virus (DENV1–4) are responsible for approximately 50 to 100 million infections annually, and 2.5 billion people are at risk of infection, making DENV the most widespread arboviral disease [1], [2]. Most symptomatic DENV infections present as a debilitating, febrile disease known as dengue fever (DF), but serious cases can progress to dengue hemorrhagic fever (DHF) and dengue shock syndrome (DSS). These can be fatal if patients do not receive fluid replacement. Currently, there are no approved therapies for DENV infections.

Clinical and autopsy studies indicate that cells of the mononuclear phagocyte lineage, including monocytes/macrophages and dendritic cells, are primary targets of DENV *in vivo* [3]. In addition, epidemiological studies show that DHF/DSS often occurs in patients with secondary heterotypic DENV infections or in infants with maternally transferred dengue immunity [4], [5]. Antibody-dependent enhancement (ADE) is thought to be a major contributor to severe disease following secondary infection [4]. In a secondary infection with a heterologous serotype, the virus forms immune complexes with pre-existing sub-neutralizing antibodies and bind to Fc-receptor-bearing cells, leading to increased infection and viral replication [5]. The cell biology of ADE is not fully understood, but some proposed mechanisms include: increased virus attachment to the cell surface, increased efficiency in post-attachment steps due to Fc-receptor-mediated signaling, delivery of antibody-virus complexes to more favorable locations in the endocytic compartment, and direct alterations in the fusion process (reviewed in [6], [7]).

We have recently shown that interferon-inducible transmembrane (IFITM) proteins restrict replication of multiple viruses, including influenza A (IAV), SARS coronavirus, filoviruses (Ebola and Marburg viruses) and flaviviruses (including dengue and West Nile viruses), whereas vesicular stomatitis virus is less efficiently restricted [8], [9]. Other groups have also demonstrated that IFITM proteins restrict HIV-1 [10], [11]. In addition, IFITM-mediated restriction of flaviviruses has been confirmed in two subsequent studies [11], [12]. The IFITM proteins are relatively small (∼130 amino acids) and share a common topology, with two conserved transmembrane domains, a short highly conserved cytoplasmic region, and luminal amino- and carboxy-termini [13]. In humans, IFITM1, 2, and 3 are expressed in a wide range of tissues, while IFITM5 expression is limited to bone [14]. As their names suggest, IFITM proteins are strongly upregulated by type I and II interferons [8], [9], [15], and most cells express basal levels of one or more of these proteins [16]. Currently, IFITM proteins are the only known mediators of innate immunity that inhibit viral infection by blocking viral entry [17].

We have demonstrated that IFITM1, 2, and 3 restrict entry mediated by IAV, SARS coronavirus and filovirus entry proteins *in vitro*, and abrogate infection by three flavivirus virus-like particles (VLPs), suggesting that IFITM proteins also restrict flavivirus infections by blocking virus entry [8]. In agreement with this, Jiang et al. [12] have shown that IFITM proteins restrict infection by flavivirus VLPs, but do not inhibit replication of flavivirus replicons. In contrast, IFITMs do not inhibit the entry processes of amphotropic mouse leukemia virus (MLV), Machupo virus (MACV), Lassa virus (LASV) or lymphocytic choriomeningitis virus (LCMV) [8].

Currently it is not clear what distinguishes the entry mechanisms of IFITM-restricted and IFITM-insensitive viruses or how IFITM proteins suppress viral entry. In addition, IFITM-mediated restriction can be bypassed by inducing viral fusion at the plasma membrane, suggesting that the site or mechanism of viral entry can affect the sensitivity to IFITM-mediated restriction [9]. Given the potential importance of ADE to severe dengue disease and the lack of full understanding of the biology of ADE, an attractive hypothesis is that, unlike direct DENV infection, ADE-mediated DENV infection bypasses IFITM-mediated restriction, thereby increasing viral replication and disease severity in a secondary infection.

In this study, we sought to determine if ADE could bypass IFITM-mediated restriction, or, alternatively, if IFITM proteins could contribute to the control of DENV in secondary infections as well. Previous studies of IFITM-mediated restriction of flaviviruses used cell lines lacking Fc receptors [8], [11], [12]. Here, we infected human myelogenous leukemia K562 cells, which bear FcγRIIa, with DENV (direct infection) or antibody-opsonized DENV (ADE-mediated infection). We show that IFITM proteins are able to restrict both direct and ADE-mediated infection, and that both infection modes appear equally sensitive to IFITM-mediated restriction.

## Results and Discussion

**Figure 1:**
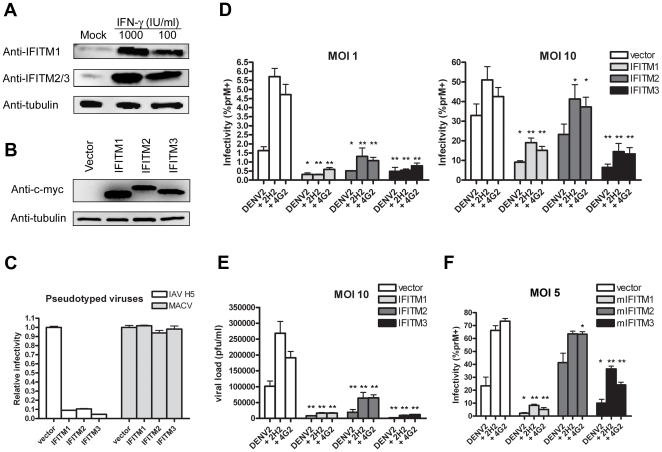
IFITM proteins restrict both direct and ADE-mediated DENV infection of K562 cells. (A) K562 cells were treated with the indicated concentrations of human interferon-α or mock treated, and lysed for Western blot analysis 48 h later, using an antibody that recognizes IFITM1 alone, or one that recognizes both IFITM2 and IFITM3. (B) K562 cells were stably transduced to express human IFITM1, 2, or 3 or with the control vector pQCXIP. IFITM protein expression was measured by Western blot using an anti-c-myc antibody. (C) Stably transduced K562 cells characterized in panel B were infected with an MLV-based retrovirus expressing EGFP and pseudotyped with MACV or IAV H5 entry proteins, as previously described [9]. Infection was determined by flow cytometry, and normalized to K562 cells transduced with vector alone. (D) K562 cells characterized in panel B were infected with infectious DENV2 NGC strain (labeled “DENV2” for virus only) at the indicated MOI, or the same amount of infectious DENV2 pre-opsonized with enhancing titers of antibodies against DENV structural proteins prM or E (“+2H2” and “+4G2” respectively) [8], [21]. Cells were washed after 1.5 h and incubated for ∼24 h. Intracellular staining of DENV antigen was performed with a DyLight-649-conjugated antibody against prM and infection was determined by flow cytometry. Experiment is representative of three with similar results. (E) An experiment similar to that shown in panel D was performed except that infection was assayed by measuring viral loads in the supernatant by plaque assays using BHK cells. (F) An experiment similar to that in panel D was performed, except that J774A.1 murine macrophage cells were stably transduced to express murine orthologs of IFITM1, 2, or 3 or with the control vector pQCXIP. Stably transduced cells were infected with infectious DENV2 NGC strain at ∼MOI 5 and incubated for ∼2 days before harvesting for flow cytometry. Error bars indicate standard error. Single and double asterisks indicate statistically significant (*P*<0.05 and *P*<0.005, respectively) differences between IFITM protein expressing and control cells for corresponding infection conditions.

We first investigated the basal and interferon-induced expression of IFITM proteins in K562 cells, using an antibody that recognizes IFITM1 alone, or one that recognizes both IFITM2 and IFITM3 [8], [9]. Western blot analysis showed that resting K562 cells express a basal level of IFITM1 and IFITM2 and/or 3, and that treatment with human IFN-α for 48 h strongly upregulated expression of both IFITM1 and IFITM2/3 (Fig. 1A).

To study the restriction activity of each IFITM protein, we transduced K562 cells to stably express myc-tagged IFITM1, 2 or 3, or with vector alone, and selected transduced cells with puromycin. Western blot analysis using an antibody against c-myc showed robust expression of each IFITM protein (Fig. 1B). To verify their activities, we infected transduced cells with viral pseudoparticles that contain an MLV genome encoding the enhanced green fluorescence protein (EGFP), and pseudotyped with entry proteins of IAV (A/Thailand/2(SP-33)/2004 (H5N1)) or MACV [18], [19]. Pseudovirus infection was measured two days later by flow cytometry. As expected, overexpression of IFITM1, 2 or 3 potently restricted infection by MLV-EGFP virus pseudotyped with IAV but not MACV entry proteins (Fig. 1C).

We next infected these cells with infectious DENV2 New Guinea C strain (NGC) propagated in mosquito C6/36 cells. DENV2 was incubated in media alone, or with enhancing titers of monoclonal antibodies against dengue prM or envelope (E). We used two commercially available antibodies, 2H2 and 4G2, which bind dengue virus structural proteins prM and E respectively. Anti-prM antibodies opsonize virions that are not fully mature while antibodies against E can opsonize most virions (reviewed in [20]). As previously described [21], dengue virions were incubated with each antibody or with media alone, and subsequently incubated with K562 cells. Previous studies have shown that intracellular staining of DENV antigen less than 43 h post-infection of K562 cells indicates a single round of infection [22]. Accordingly, to evaluate viral entry, we assessed productive infection by intracellular prM-staining 24 h post-infection [23]. As expected, with direct (antibody-independent) infection at a multiplicity of infection (MOI) 1, expression of IFITM1, 2, or 3 potently inhibited infection by ∼80% when compared to vector-transduced cells. With ADE-mediated infection at MOI 1, baseline infectivity increased roughly three-fold indicating successful ADE, but expression of IFITM1, 2 or 3 continued to potently inhibit infection by ∼70–90% (n = 3 or 4 per condition; P<0.05). Similarly, at MOI 10, we observed less efficient restriction, but comparable restriction levels in direct and ADE-mediated infection (Fig. 1D). Consistent with our previous studies, IFITM2 restricted DENV infection less efficiently than either IFITM1 or IFITM3 [8]. We further quantified viral loads in the supernatant by standard plaque assays with BHK cells [21]. Again, overexpression of IFITM1 or IFITM3 effectively restricted ADE-mediated infection, and suppressed viral production by more than 10-fold (Fig. 1E; n = 3; P<0.05). Finally, we verified the generality of our observations by confirming them in J774A.1 murine macrophages [24] stably expressing murine orthologs of IFITM1, 2 or 3 (Fig. 1F; n = 4; asterisks indicate P<0.05). We conclude that IFITM1, 2 or 3 can restrict ADE-mediated DENV infection of K562 cells.

**Figure 2:**
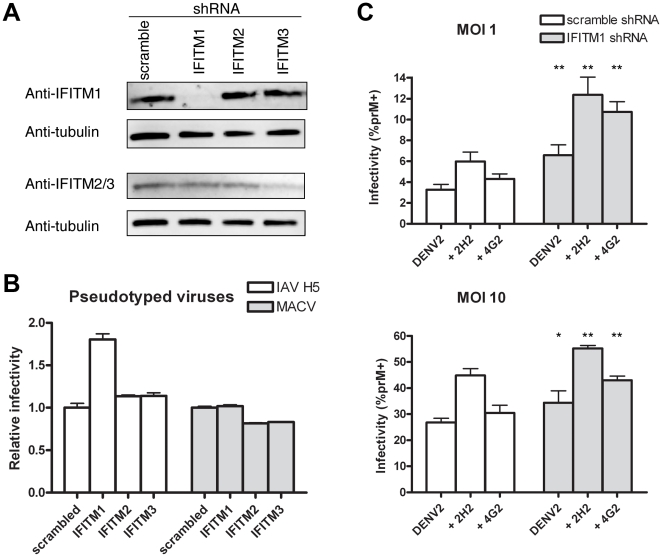
Endogenous IFITM1 restricts both direct and ADE-mediated DENV infection of K562 cells. (A) K562 cells were stably transduced to express shRNA targeting IFITM1, 2, or 3 or non-targeting control shRNA (scrambled). IFITM protein expression was measured by Western blot, as in Fig. 1A. (B) shRNA-transduced K562 cells characterized in panel A were infected with the indicated pseudoviruses as described in Fig. 1C. (C) shRNA-transduced K562 cells characterized in panel A were infected with infectious DENV2 NGC strain as described in Fig. 1D. Error bars indicate standard error. Single and double asterisks indicate statistically significant (*P*<0.05 and *P*<0.005, respectively) differences between cells expressing IFITM1 and control shRNA for corresponding infection conditions. Experiment is representative of three with similar results.

We subsequently investigated whether endogenous IFITM proteins contributed to DENV restriction. We stably transduced K562 cells to express previously characterized shRNAs against IFITM1, 2, or 3 [8], [9], or with a control non-targeting shRNA (scrambled), and selected transduced cells with puromycin. IFITM1 expression was nearly undetectable in cells expressing shRNA targeting IFITM1, whereas IFITM2 and 3 levels were unaffected. shRNA targeting IFITM2 reduced IFITM2/3 expression slightly, while shRNA targeting IFITM3 substantially reduced IFITM2/3 expression (Fig. 2A). As we have previously observed [9], IFITM1 depletion increased infection by IAV pseudoviruses by almost 2-fold, whereas little or no increase in infection was observed with shRNA targeting IFITM2 or IFITM3 (Fig. 2B). Similarly, with DENV2 infection at MOI 1, knockdown of IFITM1 roughly doubled infection rates with both direct and ADE-mediated infections (Fig. 2C; n = 3 or 4; P<0.05). This increase was less pronounced at a higher MOI of 10, but remained statistically significant. These data show that endogenous IFITM1 in K562 cells restricts ADE-mediated DENV infection.

**Figure 3:**
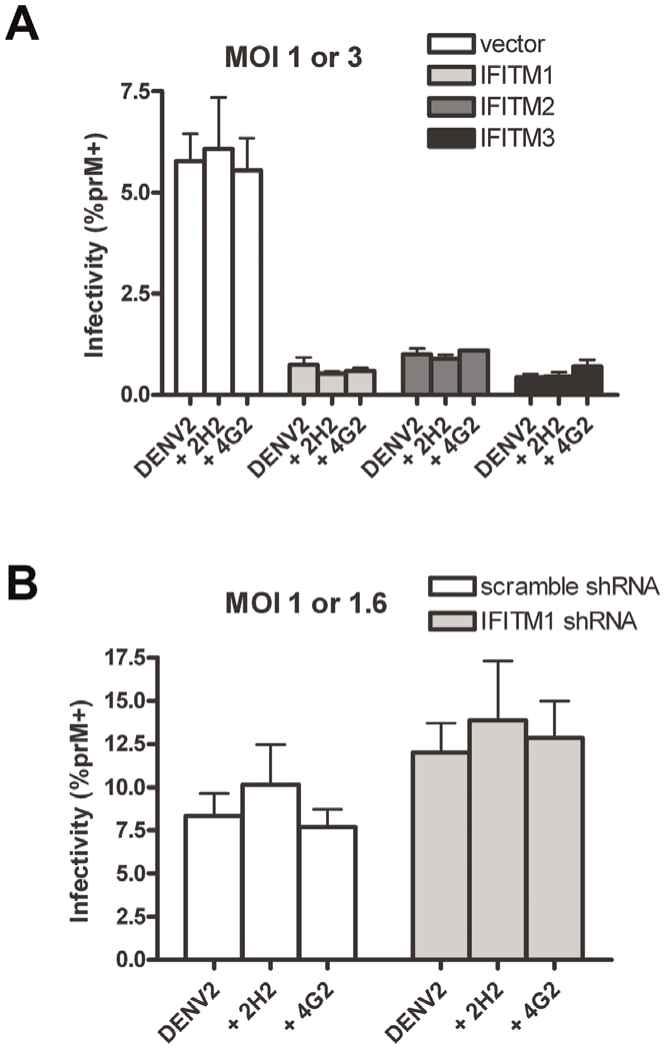
IFITM proteins restrict direct and ADE-mediated infection with similar efficiencies. (A) K562 cells stably transduced to express human IFITM1, 2, or 3 or with control vector were infected as in Fig. 1D, except that an MOI of 3.0 was used for non-opsonized DENV, and an MOI of 1.0 was used for DENV pre-opsonized with the antibodies 2H2 or 4G2. Different MOIs were used to achieve comparable infection levels between direct and ADE-mediated infection. (B) K562 cells stably transduced to express control shRNA or shRNA targeting IFITM1 were infected as described in Fig. 2C, except that an MOI of 1.6 was used for non-opsonized DENV, and an MOI of 1.0 was used for DENV pre-opsonized with the indicated antibodies. Standard error bars are shown. For each transduction condition, differences between direct infection and ADE-mediated infection were not statistically significant.

Figs. 1 and 2 show that ADE cannot bypass IFITM-mediated restriction, but leave open the possibility that ADE-mediated infection is quantitatively less sensitive to restriction than direct infection. To determine if IFITM proteins restrict ADE-mediated infection to the same extent as direct infection, we identified in pilot studies virus titers for direct and ADE-mediated infection that resulted in comparable infectivity, and used these titers to investigate the effect of IFITM overexpression or IFITM1 depletion as in Figs. 1D and 2C. When IFITM1, 2 or 3 were over-expressed in K562 cells under these conditions, nearly identical restriction was observed in direct and ADE-mediated infection (Fig. 3A). Similarly when IFITM1 was depleted in the same cells, a comparable increase in infection was observed (Fig. 3B). We conclude that IFITM proteins restrict ADE-mediated infection and direct infection with similar efficiencies.

We have previously shown that IFITM-mediated restriction depends on the site or mechanism of viral entry [9]. This finding raised a key question: can ADE facilitate bypass of this restriction, or, instead, can IFITM proteins contribute to control of a secondary infection, including those resulting in DHF/DSS? Our data show clearly that in a human myelogenous leukemia cell line widely used in the study of ADE [22], [25], [26], IFITM proteins restrict ADE-mediated infection as efficiently as direct infection. Therefore, we find no evidence supporting the hypothesis that ADE-mediated DENV infection bypasses or is less sensitive to IFITM-mediated restriction. It is formally possible that differences in cell types, antibody properties, or viral strains could alter the sensitivity of ADE-mediated infection to IFITM restriction. However we show that our observations are consistent across two different cell lines from two species expressing their respective IFITM orthologs. In addition, it has been suggested that patients with DHF exhibit lower IFN-α levels than patients with DF (reviewed in [27]), which may lead to reduced IFITM-induction. *In vivo* studies may be necessary to clarify these possibilities. Nonetheless, our observation suggests that therapeutic strategies that specifically induce IFITM activity could control both primary and secondary dengue virus infections.

To date, the mechanisms of IFITM-mediated restriction remain elusive. We have previously demonstrated that IFITM proteins do not interfere with virion access to acidic cellular compartments [9]. Furthermore, inducing viral fusion at the plasma membrane bypasses IFITM-mediated restriction [9], supporting a mechanism which operates within the endocytic pathway. Our work in this study shows that IFITM proteins restrict ADE-mediated DENV infection as efficiently as direct infection, but does not distinguish between the possible mechanisms of ADE, including alterations in viral attachment, endocytosis and/or fusion. However, the similar sensitivity of direct and ADE-mediated infection to IFITM restriction indicates that these infection modes share common features that render them both sensitive to IFITM proteins.

## Materials and Methods

### Cells and plasmids

Human embryonic kidney 293T cells and murine macrophage J774A.1 cells were grown in Dulbecco's minimal essential medium (DMEM; Invitrogen) and human myelogenous leukemia K562 cells were grown in Roswell Park Memorial Institute (RPMI) 1640 medium (Invitrogen). Media were supplemented with 10% fetal bovine serum, 100 U/ml penicillin and 100 µg/ml streptomycin. Human interferon-α (NR-3077) was obtained from NIH Biodefense and Emerging Infectious Research Resources Depository, NIAID, NIH and used to stimulate K562 cells at indicated concentrations in growth media for 48 h. Plasmids encoding c-myc-tagged IFITM proteins in pQCXIP vector and plasmids encoding control shRNA or shRNA targeting IFITMs in pRS vector have been described [9]. Transduced K562 or J774A.1 cells were selected by supplementing media with 3 or 4 µg/ml puromycin(Invitrogen), respectively.

### Western blots

Cells were lysed with 1% NP-40 (Thermo Scientific) and Western blot analysis was performed as previously described [9]. C-myc-tagged IFITM proteins were detected by a murine monoclonal anti-c-myc antibody (9E10, Santa Cruz Biotechnology). Endogenous IFITM protein expression was detected by polyclonal rabbit anti-IFITM1 (FL-125, Santa Cruz Biotechnology) or rabbit anti-IFITM2 (12769-1-AP, Proteintech Group, cross reacts with IFITM3 protein). Anti-tubulin antibodies (Sigma) were used as a loading control.

### Pseudotyped murine leukemia viruses (MLVs) for transduction and infection assays

Viral entry proteins, plasmids and procedures for generating pseudotyped MLV-EGFP have been described [9]. Entry proteins used are IAV HA proteins from A/Thailand/2(SP-33)/2004(H5N1) and glycoprotein (GP) from Machupo virus Carvallo. Similarly, transduction of K562 or J774A.1 cells and MLV-EGFP pseudovirus infection procedures have been described [9]. Relative infectivity was calculated by normalizing against infectivity in control cells.

### Infectious DENV infections

DENV2 New Guinea C strain (NGC) was propagated in *Aedes albopictus* C6/36 cells, clarified by centrifugation and stored at −80°C. Virus titers were measured by standard plaque assays on BHK cells [21]. Direct and ADE-mediated infections were performed based on a published protocol with minor modifications [21]. Murine 2H2 and 4G2 antibodies were purchased from Millipore. Briefly, DENV2 was incubated in RPMI containing 2% FBS with or without enhancing titers of 2H2 (20 ng for MOI 1 and 200 ng for MOI 10) or 4G2 antibodies (∼20 ng for both MOI 1 and 10) in a total volume of 250 µl for 30 min at 37°C. 2×10^5^ K562 cells in a similar volume of media in 24-well plates were then infected similarly to pseudovirus infection procedures. After incubation for 1.5 h at 37°C, cells were then washed in DPBS and incubated for ∼24 h before harvesting cells for flow cytometry or supernatant for plaque assay. Cells were assayed for productive infection by intracellular prM staining [23] and analyzed by flow cytometry. The supernatant was clarified by centrifugation, frozen at −80°C, before plaque assays were performed with BHK cells. The infection protocol was slightly modified for adherent J774A.1 cells. 2×10^5^ cells were seeded overnight in 12-well plates and infected with a similar total volume (500 µ l) of virus or virus-antibody complex in DMEM containing 2% FBS the next day. After infection and washing, cells were incubated for ∼2 days before harvesting for flow cytometry. Enhancing titers of 2H2 were based on a previous study [21] while enhancing titers of 4G2 were empirically determined in a pilot experiment using serial 10-fold dilutions of the antibody complexed with DENV2 at MOI 1 or MOI 10. The dilution that gave the best enhancement in infection of K562 cells was subsequently used in all experiments. All statistical analysis was performed using a one-tailed Student's *t* test. P<0.05 was considered statistically significant.
